# Silent Megacolon: Fulminant *Clostridioides Difficile* Infection Without Diarrhea Mimicking Acute Colonic Pseudo-Obstruction

**DOI:** 10.14309/crj.0000000000002149

**Published:** 2026-05-28

**Authors:** Ahmad Zain, Shafaq Saleem, Matthew Yurkewicz, Bryant Javier, Nathan Vicknair, Mohammed Mustafa Nayeem, Sung Yang, Sai Prasanna Lekkala

**Affiliations:** 1 Department of Internal Medicine, UCHealth Parkview Medical Center, Pueblo, CO; 2 Department of Internal Medicine, Rawalpindi Medical University, Rawalpindi, Pakistan; 3 Department of Gastroenterology, UCHealth Parkview Medical Center, Pueblo, CO

**Keywords:** clostridioides difficile infection, fulminant CDI, atypical CDI, toxic megacolon, acute colonic pseudo-obstruction, refractory constipation, pseudomembranous colitis, colonic dilation, stool PCR, negative toxin EIA, colonoscopy, immunocompetent patient, alcohol use disorder, septic shock, multi-organ failure, subtotal colectomy, ischemic colon, non-diarrheal CDI, diagnostic challenge, case report

## Abstract

*Clostridioides difficile* infection (CDI) typically presents with diarrhea in elderly or antibiotic-exposed patients. We report a 40-year-old immunocompetent man with alcohol use disorder who presented with abdominal pain and jaundice, rapidly progressing to refractory constipation and colonic dilation (14.7 cm) mimicking acute colonic pseudo-obstruction. Conservative management including bowel regimens and neostigmine (generally contraindicated in suspected toxic megacolon) were attempted. Colonoscopic decompression revealed cecal pseudomembranes whereas rest of colon was grossly normal. Stool testing showed discordant results that is, negative toxin enzyme immunoassay but positive stool polymerase chain reaction (Nucleic Acid Amplification Testing), supporting CDI in appropriate clinical context. Despite appropriate broad-spectrum antibiotics and emergent subtotal colectomy for fulminant disease with ischemic changes, he developed septic shock and multiorgan failure. This atypical, fulminant CDI presentation without diarrhea, initially mimicking acute colonic pseudo-obstruction, with isolated cecum and ileum involvement, underscores diagnostic pitfalls and need for early polymerase chain reaction/endoscopy in unexplained megacolon.

## INTRODUCTION

The foremost cause of healthcare-related infectious diarrhea globally is the *Clostridioides difficile* infection (CDI) secondary to antibiotic usage which disrupts the colonic microbiota, allowing unhinged *C. difficile* growth and toxin release namely Toxin A and B. This provokes mucosal inflammation, injury and ulceration and pseudomembrane formation, accounting for almost 12,800 annual mortalities in United States alone, with manifestations spanning from mild diarrheal illness to life-threatening fulminant colitis.^1^ Conventionally, the risk factors encompass advancing age, extended hospital stay, broad-spectrum antibiotics including clindamycin, fluoroquinolones, and cephalosporins as well as immunosuppressed state of patient.^2^ But interestingly, an increasing number of younger, healthier individuals without any recent illness or antibiotic exposure are being afflicted with community acquired-*Clostridioides difficile* infection (CA-CDI).^3^ *C. difficile* no longer has a diarrhea limited presentation with an increasing trend of more unusual presentations associated with constipation.^4^

The Ogilvie syndrome, also called acute colonic pseudo-obstruction (ACPO) is a form of massive non-mechanical colonic dilation, typically seen in critical hospital admitted patients.^5^ While colonic dilation secondary to toxic megacolon may be precipitated by CDI, presentation of CDI without diarrhea mimicking ACPO is exceptionally rare and diagnostically elusive. Toxic megacolon is a life threatening colitis complication, commonly associated with inflammatory bowel disease and CDI. It is defined by colonic dilation >6 cm with signs of systemic toxicity and radiographic evidence of inflammation. Contrastingly, ACPO is a functional motility disorder seen in postoperative and hospitalized patients lacking any signs of mucosal and systemic inflammation. Differentiating the 2 is critical, because the standard therapies for ACPO that is,: neostigmine and colonoscopic decompression are generally contraindicated in toxic megacolon due to the risk of perforation. Although diarrhea typically prompts CDI testing, Nwachuku et al describe a small series of CDI cases without diarrhea.^4^ We report a case of fulminant CDI in a 40-year-old man, without classic risk factors, initially presenting without diarrhea and mimicking ACPO, leading to rapid clinical deterioration.

## CASE REPORT

**Figure 1.**
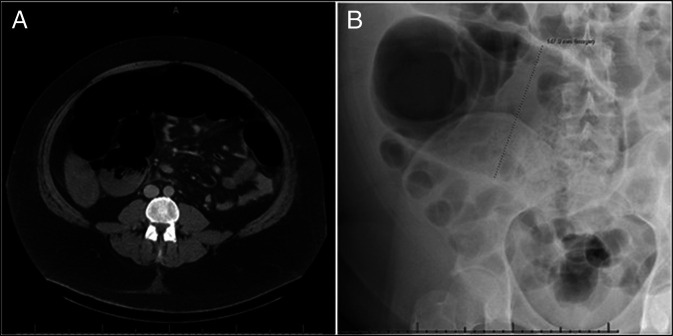
(A) CT imaging demonstrating hepatosplenomegaly only without significant colonic dilation or mural thickening. (B) CT image demonstrating abrupt segmental colonic dilation up to 14.7 cm concerning for ACPO. ACPO, acute colonic pseudo-obstruction; CT, computed tomography.

Our patient was a 40-year-old man, with alcohol use disorder and seizures history. He presented to us with abdominal pain, jaundice, and deranged liver function tests such as aspartate aminotransferase peaking at ∼ 3,492 U/L (N: 10-49 U/L), alanine aminotransferase at 727 U/L (N: 7–56 U/L), bilirubin between 2.3 and 2.7 mg/dL (N: 0.1–1.2 mg/dL). Alkaline phosphatase ∼ 160 (N: 44–147 U/L), and albumin 2.6 g/dL (N: 3.5–5.0 g/dL). Initial workup focused on hepatobiliary causes like acute pancreatitis, choledocholithiasis or ischemic vs alcoholic hepatitis but his abdominal computed tomography showed hepatosplenomegaly without any evidence of pancreatitis, cholecystitis or cirrhosis, significant colonic dilation or mural thickening as shown in Figure 1.

Within 48 hours of his hospitalization, the patient developed refractory constipation with progressive abdominal distention. Repeat imaging showed abrupt segmental colonic dilation upto 14.7 cm concerning for ACPO also called the Ogilvie Syndrome, as shown in Figure 1.

**Figure 2.**
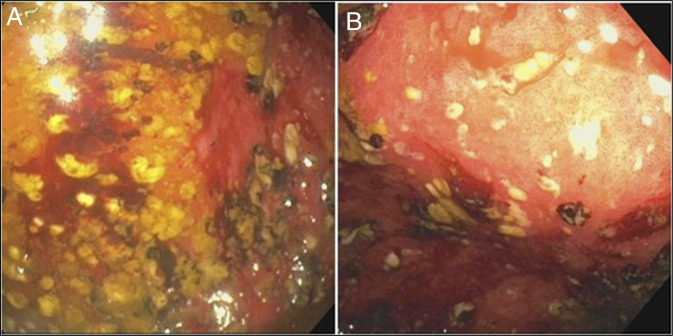
(A) Colonoscopic image demonstrating diffuse yellow-white pseudomembranes overlying erythematous and friable mucosa in the cecum, consistent with pseudomembranous colitis. These plaques represent necrotic epithelial debris, fibrin, mucus, and inflammatory exudate characteristic of fulminant *Clostridioides difficile* infection. (B) Colonoscopic image showing patchy pseudomembranous involvement with adjacent relatively preserved mucosa, highlighting the segmental (ileocecal-predominant) distribution of disease in this case, which is atypical compared with the usual diffuse colonic involvement seen in *Clostridioides difficile* colitis.

The patient was started on conservative measures including nil per mouth, bowel rest, enemas, bowel regimens, and intravenous neostigmine. An attempt at therapeutic colonoscopic decompression was made due to persistent abdominal distention. Endoscopic evaluation revealed pseudomembranes carpeting the cecal mucosa; whereas the remaining colon was grossly unremarkable as shown in Figure 2.

Histopathologic examination of cecal biopsy specimens demonstrated nonspecific mucosal injury with a differential diagnosis including ischemic vs infectious colitis.

Given the endoscopic findings, stool testing for CDI was performed. Stool enzyme immunoassay for the toxin came back negative, but Nucleic Acid Amplification Testing (NAAT) was positive.^6^ In context of pseudomembranes and clinical deterioration, these findings were interpreted as consistent with CDI and targeted antimicrobial therapy was initiated with vancomycin and metronidazole. Despite treatment, the patient's condition deteriorated rapidly with fulminant CDI along with progressively worsening leukocytosis (white blood cell >30,000/µL), lactic acidosis (lactate 12 mmol/L), and altered mental status leading to septic shock and acute respiratory distress syndrome, which required intubation and vasopressor support. Fidaxomicin was added, but he continued to decline.

**Figure 3.**
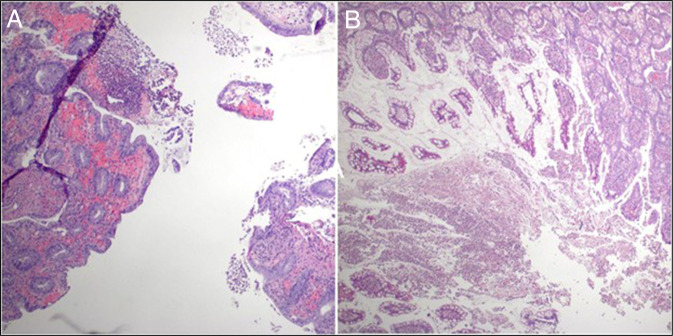
(A) Histopathology demonstrating congested blood vessels are also present in the submucosa (H&E, 40×). (B) Histology demonstrates ileal mucosa with superficial ulceration. Pseudomembranes are composed of necrotic debris, apoptotic epithelial cells, inflammatory cell (neutrophilic) exudate and mucus (H&E, 200×). H&E, hematoxylin and eosin.

An emergent subtotal colectomy was performed due to fulminant clinical decline, which unveiled grossly ischemic gangrenous colon intraoperatively. The histopathologic evaluation of resection specimen revealed focal acute ileitis and colitis with pseudomembrane formation and prominent vascular congestion and lamina propria edema as shown in Figure 3 without evidence of any malignancy. The vermiform appendix was benign.

Ultimately, despite rigorous medical and surgical interventions, he developed refractory hypotension and profound acidosis leading to death.

## DISCUSSION

Our case challenges the conventional epidemiological profile of CDI on multiple fronts such that; first, the classic textbook cases include elderly, immunosuppressed, and antibiotic-exposed patients. By contrast, our patient was a 40-year-old, immunocompetent young man without any recent antibiotic exposure. His only notable risk factor was alcohol use disorder. The chronic alcoholism impairs neutrophil function, T-cell immunity and gut barrier integrity, creating a portal for bloodstream entry of pathogens.^7^ This supports maintaining suspicion for CA-CDI in younger patients without traditional healthcare exposure. Second, the most significant misdirection was the complete absence of diarrhea. The presence of profuse diarrhea in patients typically triggers early CDI testing. We hypothesize that the toxin mediated neural inhibition and transmural inflammation resulted in colonic dysmotility and dilation leading to constipation, which clinically mimicked ACPO.^8^ Despite its clinical resemblance to ACPO, the patient's systemic toxicity and mucosal inflammation indicated toxic megacolon rather than a purely functional disorder. Importantly, the diagnostic delay occurred because of the absence of diarrhea as clue leading to late CDI focus. Recent reviews highlight increasing recognition of atypical and community-acquired CDI presentations, including cases without diarrhea and those mimicking ileus or pseudo-obstruction.^9^

A critical diagnostic challenge in this case was the discordance between stool assays, with a negative toxin enzyme immunoassay and positive NAAT. According to the Infectious Diseases Society of America 2021 guidelines, NAAT alone cannot distinguish between active infection and asymptomatic colonization, as it detects the presence of toxigenic *C. difficile* genes rather than active toxin production. Therefore, reliance on polymerase chain reaction alone may lead to overdiagnosis in the absence of compatible clinical findings. However, in this patient, multiple features strongly supported true fulminant infection rather than colonization, including: (i) direct endoscopic visualization of pseudomembranes, which are highly specific for *C. difficile* colitis, (ii) marked systemic toxicity with leukocytosis (>30,000/µL), lactic acidosis, and shock, (iii) progressive colonic dilation with ischemic changes, and (iv) histopathologic confirmation of pseudomembranous inflammation in resected specimens. Current guidelines emphasize that diagnosis should integrate clinical presentation, laboratory testing, and endoscopic or histologic findings, particularly in atypical cases. In this context, the overall clinicopathologic image was most consistent with fulminant CDI, despite toxin assay negativity.^10^

And finally, per the management and prognosis, neostigmine was attempted due to initial ACPO suspicion to target dysmotility.^11^ Neostigmine is generally avoided in toxic megacolon due to perforation risk. Similarly full colonoscopy also carries significant risk and only flexible sigmoidoscopy with biopsy is recommended when endoscopic evaluation is necessary.^12^ Once diagnosed, vancomycin and metronidazole were started, but cytokine storm and bacterial translocation had begun. Subtotal colectomy, indicated for refractory shock or ischemia, was performed, but preoperative lactic acidosis (>10 mmol/L), vasopressor need, and multiorgan failure predicted poor outcome. Mortality after colectomy for fulminant CDI is 40%–50%, rising sharply with delayed surgery.^13^ An additional unique feature in this case was predominant ileocecal distribution of disease initially. CDI most often involves colon diffusely. Predominant ileocecal involvement, as seen in this case, is rare and has been described mainly in fulminant diseases or partially treated infections. The surgical resection specimen showing focal ileocecal and colonic involvement supports partial progression rather than the classic pancolitis. This also highlights the dynamic nature of fulminant CDI, which may begin with segmental disease and later extend to other colonic regions.

Severe colonic dilation and toxic megacolon may co-occur with CA-CDI, even in younger individuals without the traditional risk factors. Although a single case cannot prove causation, our case report underscores the importance of suspecting CDI in patients with unexplained colonic distention. Early stool testing should be performed using recommended 2-step algorithms. Endoscopic evaluation should be performed in cases of constipation where a stool sample is not available or if clinical suspicion remains high despite negative stool-based testing. This case reminds clinicians that CDI can have atypical presentations and that awareness of these rare forms may support earlier recognition and timely management and can reduce the risk of mortality.

## DISCLOSURES

Author contributions: A. Zain: Conceived the case report, contributed to patient management, data acquisition, and initial manuscript drafting. S. Saleem: Conducted literature review, refined the case description, and prepared the final manuscript draft. M. Yurkewicz: Provided gastroenterology expertise, contributed to diagnostic interpretation, and critically reviewed the manuscript. B. Javier: Contributed to endoscopic assessment details, provided clinical insights, and reviewed the manuscript for accuracy. N. Vicknair: Supported gastrointestinal evaluation, contributed to data interpretation, and revised the manuscript critically. MM Nayeem: Assisted with clinical data interpretation, contributed to the discussion section, and critically revised the manuscript. S. Yang: Oversaw the clinical aspects of the case, ensured accuracy of findings, and provided final critical review and approval of the manuscript. SP Lekkala: Assisted in patient management, data verification, and critical revision of the manuscript for important intellectual content. A. Zain is the article guarantor.

Financial disclosure: None to report.

Previous presentation: Presented as an abstract at the American College of Gastroenterology Annual Scientific Meeting, October 24–29, 2025, Phoenix Arizona.

Informed consent was obtained for this case report.

## ABBREVIATIONS:

CDI: *Clostridioides difficile* infection
